# Chemi-Net: A Molecular Graph Convolutional Network for Accurate Drug Property Prediction

**DOI:** 10.3390/ijms20143389

**Published:** 2019-07-10

**Authors:** Ke Liu, Xiangyan Sun, Lei Jia, Jun Ma, Haoming Xing, Junqiu Wu, Hua Gao, Yax Sun, Florian Boulnois, Jie Fan

**Affiliations:** 1 Accutar Biotechnology Inc., 760 Parkside Ave., Brooklyn, NY 11226, USA; 2 Amgen Inc., 1 Amgen Center Dr., Thousand Oaks, CA 91320, USA; 3 Amgen Inc., 360 Binney St., Cambridge, MA 02141, USA

**Keywords:** deep learning, ADME prediction, drug discovery

## Abstract

Absorption, distribution, metabolism, and excretion (ADME) studies are critical for drug discovery. Conventionally, these tasks, together with other chemical property predictions, rely on domain-specific feature descriptors, or fingerprints. Following the recent success of neural networks, we developed Chemi-Net, a completely data-driven, domain knowledge-free, deep learning method for ADME property prediction. To compare the relative performance of Chemi-Net with Cubist, one of the popular machine learning programs used by Amgen, a large-scale ADME property prediction study was performed on-site at Amgen. For all 13 data sets, Chemi-Net resulted in higher R^2^ values compared with the Cubist benchmark. The median R^2^ increase rate over Cubist was 26.7%. We expect that the significantly increased accuracy of ADME prediction seen with Chemi-Net over Cubist will greatly accelerate drug discovery.

## 1. Introduction

The four essential processes of drug absorption, distribution, metabolism, and excretion (ADME) all influence the performance and pharmacological activity of potential drugs. Over the years, the experimental ADME properties of many compounds have been collected by the pharmaceutical industry, which have been used to predict the ADME properties of new compounds. As such, ADME property prediction can be particularly useful in the drug discovery process to remove compounds which are more likely to have ADME liabilities during downstream development.

Inspired by the huge success of deep neural networks (DNNs) in computer vision, natural language processing, and voice recognition, and based on their remarkable capability of learning concrete and sometimes implicit features [1], we hypothesized that DNNs could be used in drug ADME property prediction. In this paper, we extend the use of traditional statistical learning methods and construct a multi-layer DNN architecture, named “Chemi-Net,” to predict the ADME properties of molecule compounds.

Applying DNNs to the prediction of ADME properties was previously reported by Ma et al. [2], Kearns et al. [3], and Korotcov et al. [4], who all demonstrated accuracy improvements with DNNs over other traditional machine learning methods. However, the core challenge of ADME prediction using DNNs is that unlike images, which can usually be represented as a fixed-size data grid, molecular conformations are generally represented by a graph structure. This structured format is heterogeneous among molecules, which is a major problem for many learning algorithms that expect homogeneous input features. Several methods have been developed to alleviate this problem. Previous research mainly focused on transforming the graph structure of molecules to a fixed size of feature descriptors. These descriptors can then be easily used by existing machine learning algorithms. Another method, which is popular, is the use of molecular fingerprints, such as those used in the Extended-Connectivity Fingerprints (ECFP) method [5]. This method encodes the neighboring environment of heavy atoms in a compound to a hashed integer identifier, with each unique identifier corresponding to a unique compound substructure. Using this method, a compound is described as a fixed-length bit string, with each bit indicating whether a certain substructure is present in the compound. Such fingerprint-based representation makes learning graph-structured molecules possible. Neural network-based methods with fingerprint inputs have also been developed following recent advances in deep learning techniques, which have been shown to significantly improve on current Random Forest-based models [2]. However, the fingerprint-based method suffers from a fundamental issue in that the space required for fingerprints can be very large. Hence, the resulting fingerprints are very sparse. Also, the information that fingerprints encode is noisy. Consequently, these factors limit the performance of fingerprint-based representations.

Recently, there has been a growing interest in using neural networks to directly obtain a representation of a compound ligand before applying other layers of neural network to build the predictive models. These methods transform a molecule to a small and dense feature vector (embedding), which is easier for downstream learners to use. These methods use string-based representation of molecules [6], a graph convolution architecture to model circular fingerprints [1], and also the Weave module in which atom and pair features are combined and transformed through convolution-like filters [7].

Studies to date, which have applied DNNs to ADME predications, have shown that multi-task deep neural networks (MT-DNNs) have advantages over traditional single-task methods [2,3]. For example, MT-DNNs take advantage of neural networks’ ability to allow use of a combinational model, which has predictive power for multiple activities, being simultaneously trained with data from different activity sets. The enhanced predictive power of MT-DNNs had not been clearly explained until Xu et al. [8] found that a MT-DNN borrows “signal” from molecules with similar structures in the training sets of the other tasks. They also found that MT-DNN outperforms the single-task method if the different data sets share certain connections, and the activities across different sets have non-random patterns.

The potential application of MT-DNNs in pharmaceutical drug discovery was reviewed by Ramsundar et al. [9]. In their review, the authors confirmed the robustness of MT-DNNs and also suggested that MT-DNNs should be combined with advanced descriptors, for example, descriptors developed by graph convolutional methods to enhance the performance of a MT-DNN.

Our current application features a molecular graph convolutional network combined with the MT-DNN method to further boost prediction accuracy. To the best of our knowledge, there are no published studies that have used these combined methods. In addition, Chemi-Net implements a novel dynamic batching algorithm (described in the Supplementary Figure S1) and a fine-tuning process to further improve the stability and performance of trained models. In this study, a large-scale ADME prediction test was carried out in collaboration with Amgen. The test involved five different ADME tasks with over 250,000 data points in total. The test was conducted in a restricted environment so that the evaluation was only carried out once on the testing dataset. Our findings showed significant performance advantages with Chemi-Net over existing Cubist-based methods.

## 2. Results

### 2.1. MT-DNN Method of Chemi-Net Improves Predictive Accuracy Comparing to Cubist

**Figure 1:**
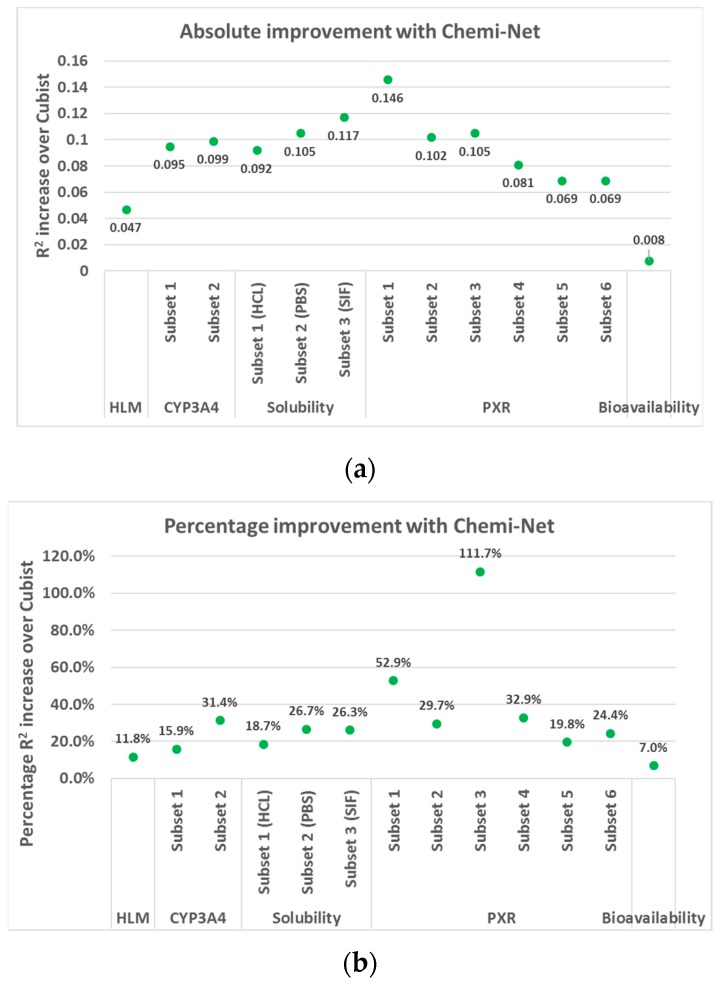
Absolute (**a**) and percentage (**b**) R^2^ improvement over Cubist using Chemi-Net. CYP3A4, cytochrome P450 3A4; HLM, human microsomal clearance; PXR, pregnane X receptor.

A large-scale test was performed on Amgen’s internal data sets using five ADME endpoints, with a total of 13 data sets selected for testing. See Section 4.8 for a detailed description of the data set. Table 1 and Figure 1 show the overall test set prediction accuracy comparison between Chemi-Net and Cubist. Performance of models developed by different algorithms is highly dependent on size of data set, type of endpoint, type of model, and molecular descriptors used. For all 13 data sets, Chemi-Net resulted in higher R^2^ values compared with the Cubist benchmark. With the single-task method, larger and less noisy data sets yielded higher improvements than the smaller and noisier data sets. Additional accuracy improvement was further achieved with MT-DNN.

**Figure 2:**
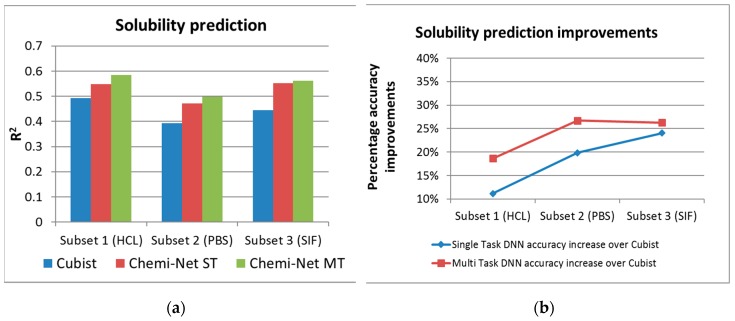
Absolute (**a**) and percentage (**b**) of R^2^ increase between ST-DNN and MT-DNN and the Cubist benchmark for the solubility endpoint. DNN, deep neural network; HCl, hydrochloric acid; MT, multi-task; PBS, phosphate-buffered saline; SIF, simulated intestinal fluid; ST, single task.

**Figure 3:**
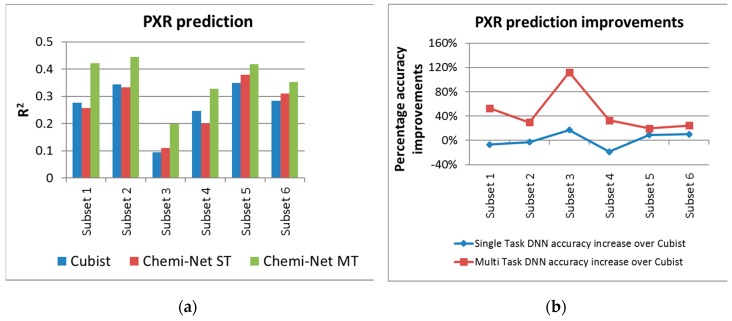
Absolute (**a**) and percentage (**b**) of R^2^ increase between ST-DNN, MTT- DNN and the Cubist benchmark for the PXR inhibition endpoint. DNN, deep neural network; MT, multi-task; PXR, pregnane X receptor.

Multi-task prediction was carried out on the solubility and PXR inhibition rate data sets, as they had multiple subsets with large and balanced training data. Figure 2 and Figure 3 show the absolute and percentage R^2^ increase between ST-DNN, MT-DNN and the Cubist benchmark for solubility, and PXR inhibition, respectively. For the lower quality PXR data set, ST-DNN had lower R^2^ values compared to Cubist for some subsets. In contrast, MT-DNN demonstrated significant improvement in R^2^ values and had higher accuracy versus Cubist for all data sets analyzed.

### 2.2. Prediction Performance and Compound Similarity

We hypothesized that, as with traditional machine-learning methods, deep learning performance is affected by similarities between the training set and test set. To investigate this further, the similarity of compounds within the training set and the similarity between training and test sets were calculated. Similarity was calculated using molecular fingerprints and the Tanimoto method [10]. The prediction models were challenged by the test sets, which contained newer compounds and novel chemotypes. For all 13 data sets, the average similarity within training sets was 0.878, and the average similarity between training and test sets was only 0.679.

**Figure 4:**
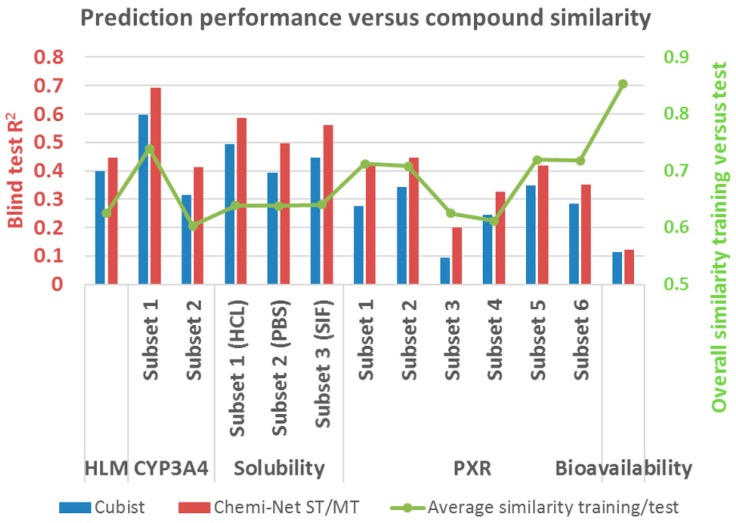
Prediction performance and similarity between training and test sets for all 13 data sets. HLM, human microsomal clearance (HLM); HCl, hydrochloric acid; PBS, phosphate-buffered saline; pregnane X receptor; SIF, simulated intestinal fluid; ST, single-task; MT, multi-task.

**Figure 5:**
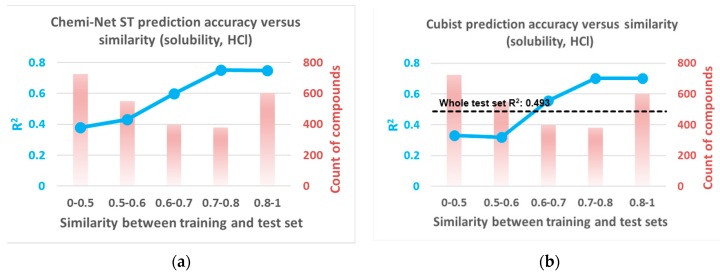
Prediction performance of binned test set compounds by similarity for the solubility (HCl) data set: Chemi-Net ST-DNN (**a**) and Cubist (**b**). HCl, hydrochloric acid.

Figure 4 shows the prediction performance in comparison to the similarity between training and test sets. In the same type of assay (e.g., solubility or PXR), prediction performance correlated with the overall compound similarity between training and test sets for both Cubist and Chemi-Net. To further illustrate the similarity influence of prediction accuracy, one data set was chosen, solubility (HCl), and the compounds in the test set were binned based on their similarity to the training set. The binned compounds were then correlated with their prediction accuracy (Figure 5). Unsurprisingly, the R^2^ increased as the similarity between the training and the test sets increased with both the Chemi-Net and Cubist models. This result strongly suggests our hypothesis is correct.

### 2.3. Comparison between Chemi-Net’s Descriptors and Amgen’s Traditional Property and Molecular Keys Descriptors

**Figure 6:**
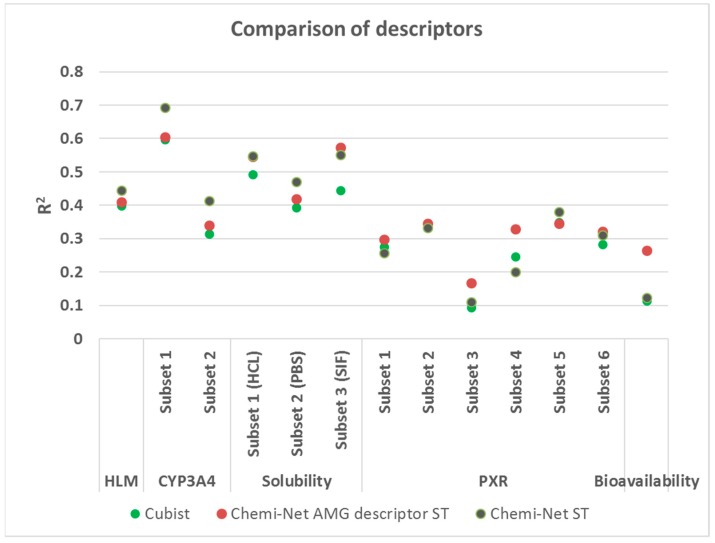
Comparison between Chem-Net molecular graph convolutional network derived descriptors and traditional descriptors. CYP3A4, cytochrome P450 3A4; human microsomal clearance (HLM); HCl, hydrochloric acid; PBS, phosphate-buffered saline; PXR, pregnane X receptor.

Chemi-Net applies molecular graph convolutional networks to generate descriptors on a three-dimensional (3D) level based on simplified molecular-input line-entry system (SMILES) strings. Over the past 10 years, Amgen has used a set of 800 more “traditional” one-dimensional (1D) and two-dimensional (2D) descriptors based on physical properties, molecular keys, etc. In our current study, we compared the two sets of descriptors by using the same ST-DNN methods in Chemi-Net (Figure 6). Interestingly, the Amgen “traditional” descriptor set and the Chemi-Net descriptor sets performed similarly in some data sets (i.e., solubility (HCL and SIF), PXR (Subset 2, 5, and 6)). For large and relatively high-quality data sets (e.g., HLM, CYP3A4), Chemi-Net descriptor sets performed better than the Amgen descriptor set. In contrast, for small and noisy data sets (e.g., PXR and bioavailability), the Amgen descriptor set performed better.

### 2.4. Chemi-Net’s Performance on Public Data Sets

In this section, we report the performance of Chemi-Net on public data sets to allow comparison with other methods. These data sets reported by Wenzel et al. [11] for metabolic clearance and passive permeability in Caco-2 cells were extracted from ChEMBL [12,13] v23. Table 2 summarizes the performance of Chemi-net and Wenzel et al.’s method on these data sets.

The results agree with trends discovered in testing Amgen’s internal data set. For the larger three data sets, Chemi-Net’s graph convolution structure shows superior performance than using traditional descriptors. Chemi-Net’s performance is low in the mouse microsomal clearance dataset, which we hypothesize is due to the small size of the training and testing sets, causing the neural network to overfit.

### 2.5. Industrial Implementation of Chemi-Net for ADME Prediction

**Figure 7:**
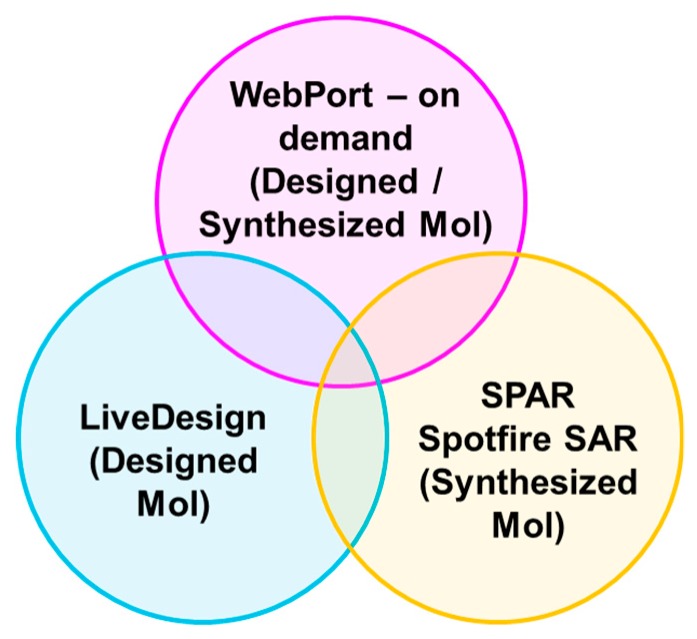
Three in silico drug design platforms used in Amgen’s Pipeline Pilot Webport for on-demand absorption, distribution, metabolism, and excretion (ADME) prediction of designed or synthesized compounds. LiveDesign by Schrodinger for ADME prediction of designed compounds. Spotfire Project Analysis Report (SPAR) for ADME prediction of synthesized compounds.

After obtaining solid accuracy performance, as well as robust operation of Chemi-Net, we decided to apply this application to Amgen’s small molecule drug discovery pipeline. We have confidence in Chemi-Net’s deep learning performance for ADME properties, which have large data sets and can be multi-task learned (e.g., solubility, liver microsome clearance etc.). These ADME properties are the first data sets that we wanted to implement. We have an existing Pipeline Pilot platform, which has been used for providing ADME prediction service for more than 20 properties. Our goal was to apply the deep learning based ADME prediction to our three in silico drug design platforms (Figure 7) and make it work seamlessly with our existing ADME prediction service (non-deep learning). We developed a training module and a prediction module in Pipeline Pilot. The training module takes advantage of the incremental training feature of Chemi-Net. It runs on a weekly basis to refresh models with newly measured data from experiments. The prediction module can be run “on-the-fly” based on the ADME prediction demand. The challenges of implementation work are the following: Heavy computing with on-demand prediction request; large data set assembly and processing; incorporating the module into Amgen’s existing ADME prediction service with traditional machine learning methods.

#### 2.5.1. Heavy Computing with on Demand Prediction Request

**Figure 8:**
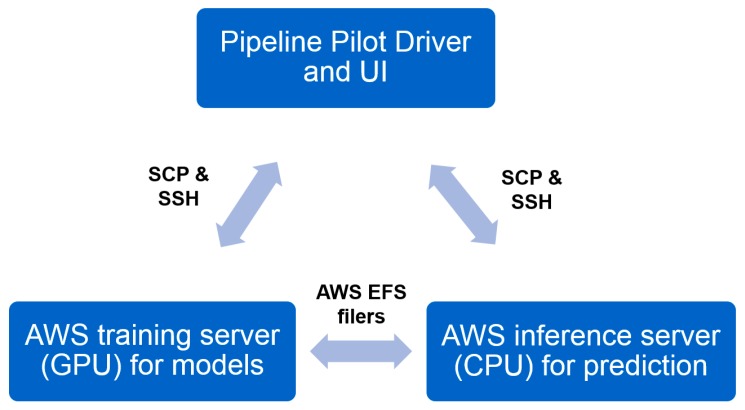
Relationship between the local Pipeline Pilot application and remote servers on Amazon Web Services (AWS).

To be able to meet the heavy computing demand required, we utilized the cloud computing resource, Amazon Web Services (AWS). Two servers on AWS were set up. The training server is a GPU instance (either p2.8xlarge or p3.8xlarge, depending on availability) for model building/refreshing. This training server is called by the training module in Pipeline Pilot. The inference server is a CPU instance (m5.4xlarge) for prediction. The inference server is called by both training and prediction modules. Pipeline Pilot SSH and SCP components are used to communicate to the remote server for driving the computation, as well as data transfer (Figure 8). An Elastic File System (EFS) storage system was setup and mounted on both AWS servers to host the trained models. The models were also backed up at our local data center. To maintain the robustness of network connection, Elastic IPs were setup and applied on both servers so that they can be accessed with a constant IP.

#### 2.5.2. Large Data Set Assembly and Processing

**Figure 9:**
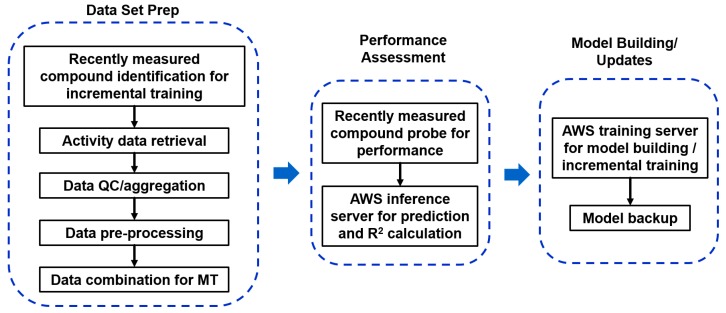
Training module setup.

A key part of our training module is designed to deal with data set challenges (Figure 9). Large data sets were used to train prediction models with MT learning. For example, the solubility model is trained with three data sets, each containing more than 10,000 data points. These data were retrieved from our Assay Data Warehouse (ADW) and compound registration database via SQL queries. A quality check mechanism was implemented to allow us to validate and clean the data, which is suitable for training calculation without errors. Non-numerical data were removed. Quantifiers (<, >, ≃) in front of numerical data were also removed. Mean values were taken for duplicated data with the same compound. We also applied log10 or logit transformation as the pre-processing step before training. In the incremental training case, which is the most commonly used to refresh our ADME models, we set up a data comparison system that can identify the new training data from the old training data, which were used to train the model. Finally, the data from different sets were assembled together to produce a simple comma delimited file.

#### 2.5.3. Incorporation to Amgen’s Existing ADME Prediction Service

We used the same Pipeline Pilot platform used previously to incorporate deep learning based ADME prediction seamlessly with Amgen’s current ADME prediction service. For the prediction module, we developed additional Pipeline Pilot components with the same data input and output format as the existing ADME application. The new components were inserted into the existing ADME application. A logic switch was built into the application to decide whether to use the deep learning-based model or existing model, based on specified ADME properties. Our existing graphic user interface for ADME prediction does not need to be modified. As the final result, the users of ADME service do not see any difference in terms of their input options as well as the output they obtain.

#### 2.5.4. Additional Features

The accuracy performance of the deep learning method is important to us. We set up a mechanism in the training module to allow us to live-check the performance during model update and archive the accuracy in R^2^, standard error, as well as the percentage of compounds being accurately predicted within two or three folds of experimental results. During the model update run, we retrieved new molecules with newly measured data since the previous training. We then used the model from the last training process to predict the assay activity and compared it to the experimental value to calculate R^2^, as well as other performance metrics (Figure 9). In this case, we made sure the new molecules are not present in the last training model used for this evaluation. The predicted and experimental values were also archived for future cumulative performance analysis.

**Figure 10:**
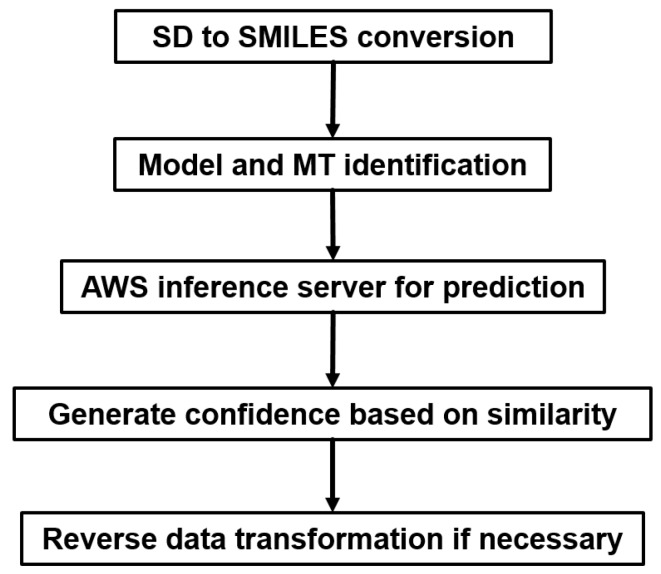
Prediction module setup.

In the prediction module, we also implemented a prediction confidence score (Figure 10). This score is based on the Tanimoto similarity between the input molecule and the molecules in the training set. We used an assumption that in machine learning methods, the prediction accuracy, or confidence positively correlates to the molecule similarity to the training data. In addition, we also output the most similar molecules in the training set to provide chemists insights for chemists.

## 3. Discussion

In this proof-of-concept study, we report the application of a molecular graph convolutional network combined with the MT-DNN method (Chemi-Net) to predict drug properties in a series of industrial grade data sets for the first time. The major improvements of this method are two-fold. First, instead of relying on preset descriptors (features) as reported in previously reported studies [2], it used a graph convolution method to extract features from the SMILE file of each compound. We compared the results with Amgen’s “traditional” descriptor set and found that while the gain from graph convolution is generally positive, for some datasets (e.g., PXR and bioavailability), the Amgen descriptor set performed better. We also hypothesize this is due to the fact that these data sets are small and noisy, which causes the DNN to be unable to learn a good representation of the data points and overfit the training data. To overcome this issue, we propose the second improvement, the MT-DNN method, which uses multitask learning to use information in large datasets to compensate for small data sets. Experiments show that this greatly improves the predictive power of the DNN model in both large (solubility) and small (PXR) datasets.

In addition to these improvements, we used a model ensemble procedure (Section 4.5) to fine-tune the performance of the neural network model. The combination of these techniques shows greater gains over prior research in which only a single technique was applied [2,11,13,14,15,16]. An interesting line of future work is to combine the automatically learned graph convolution descriptors with the handcrafted descriptors. This should help the DNN to learn a better data representation when the size of the dataset is small. Given the clear performance improvement across all assay types, we expect the wider application of our novel approach in drug discovery tasks.

## 4. Materials and Methods

### 4.1. Deep Neural Network-Based Model

**Figure 11:**
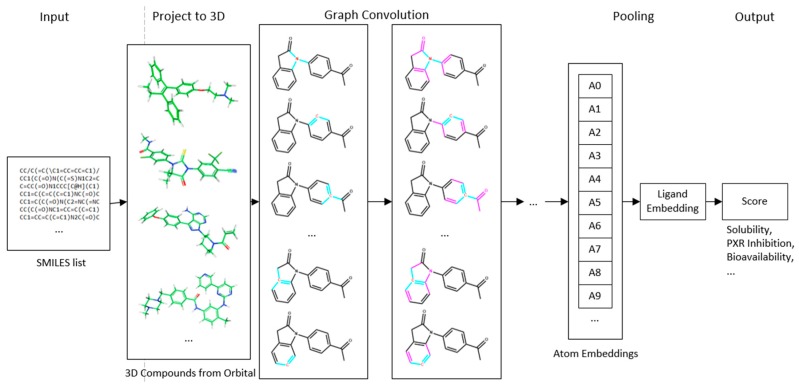
Overall network architecture. The input is quantized as molecule-shaped graph structure. Then, a series of graph-based convolution operators are applied. In this figure, red highlights the central atom, and cyan highlights concerned neighbor atoms. In the second convolution layer, purple also highlights the information flow area from the previous convolution step.

Conventional fingerprint and pharmacophore methods usually require that explicit features are extracted and trained, hence the forms of the fingerprints are often limited by human prior knowledge. Encouraged by recently reported studies in which DNNs have been shown to surpass human capability in multiple types of tasks from pattern recognition to playing the game Go [17], we decided to use a DNN architecture to develop an ADME property prediction system. The overall neural network architecture is shown in Figure 11. This network accepts a molecule input with given 3D coordinates of each atom. It then processes the input with several neural network operations and outputs the ADME properties predicted for the input molecule.

In the model presented in this paper, the input molecule is represented by a set of distributed representations assigned to its atoms and atom pairs. Both the atom and atom pair are assigned a dense feature map, with $A_{a}$ defined as the feature map of atom a, and $P_{a,b}$ defined as the feature map of atom pair a and b. Typical atom features include atom type, atom radius, and whether the atom is in an aromatic ring. Typical atom pair features include the inter-atomic distance and the bond orders between two atoms. The input molecule is then represented by a set of atom features $\left\{A_{1},A_{2},\ldots ,A_{n}\right\}$ and atom pair features $\left\{P_{a,b}|b\in neighbor\left\{a\right\}\right\}$, where a is a central atom.

After the input atom level and atom pair level features are assembled, they are combined to form a molecule-shaped graph structure. A series of convolution operators are then applied to the graph, which transforms the atom feature maps. To enable position invariant handling of atom neighbor information, the convolution filters for all atoms share a single set of weights. The output of the convolution layers is a set of representations for each atom. The pooling step reduces the potentially variable number of atom feature vectors into a single fixed-sized molecule embedding. The molecule embedding is then fed through several fully connected layers to obtain a final predicted ADME property value.

### 4.2. Convolution Operator

**Figure 12:**
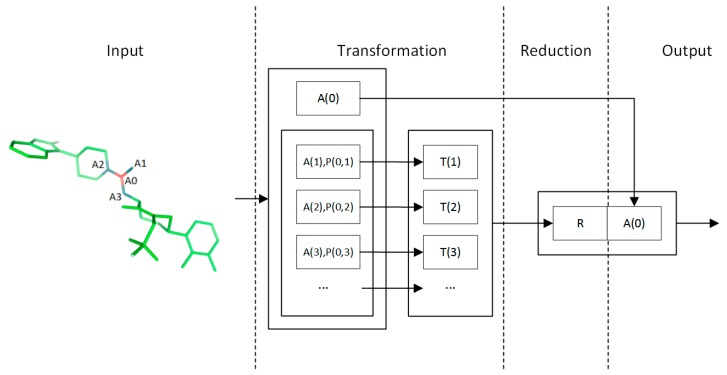
Convolution filter structure.

The convolution operator is inspired by the Inception [18] and Weave modules [7]. The overall convolution operator structure is depicted in Figure 12. The inputs of this operator are the feature maps of the atoms and atom pairs. In this operator, the feature map of each atom is updated by first transforming the features of its neighbor atoms and atom pairs, then by reducing the potentially variable-sized feature maps to a single feature map using a commutative reducing operator. Importantly, atom pair features are never changed throughout the process.

The method used to update the feature map of each atom is the same for all atoms. They are formulated with shared weights to achieve position-invariant behavior. Hence, this process can be viewed as the same convolutional operation seen in convolutional neural networks (CNNs), except that the convolution filter connections are dynamic instead of fixed. This operator is designed so that an arbitrary number of these operators can be stacked. As in DNNs, the increased number of stacking operators enables more complex structures of the molecule to be learned. A typical computation flow of a convolution filter is shown in Figure 12. The most important aspects of the filter are the transformation and reduction operators.

In the transformation step, feature maps of neighbors of an atom are transformed by a feed-forward sub-network. For a neighbor atom b of central atom a, the input feature map is the concatenation of atom feature $A_{b}$ and the atom pair feature $P_{a,b}$. The bias term is denoted as B. The input is transformed through one fully connected layer and a non-linearity function f:

$$T_{a,b}^{k}=f\left(W^{k}\left(Concat\left\{A_{b}^{k},P_{a,b}\right\}\right)+B^{k}\right)$$

After each neighbor atom of a is transformed, these feature maps are then aggregated and reduced to a single feature map. In this process, a commutative reduction function is used to keep the order-invariant nature of the input feature maps. A typical example of such a function is the element-wise sum function Sum{⋅}, which for input vectors $X_{1},X_{2},\ldots ,X_{n}$, the output vector Y is defined as $Y_{j}=\sum_{i}^{n}X_{ij}$. Similarly, we define operator Max{⋅} for element-wise max and Avg{⋅} for element-wise averaging.

**Figure 13:**
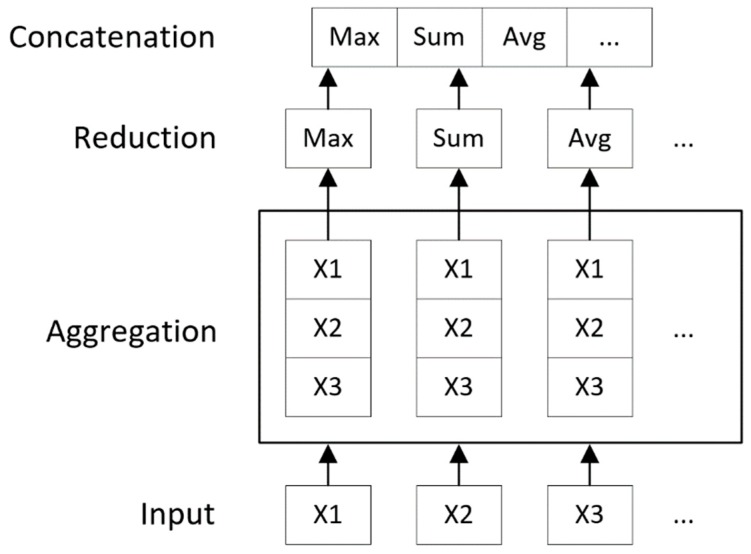
The reduction step in convolution filter. Max, maximum; Avg, average.

Following these principles, a reduction operator is constructed to improve model quality, in which multiple kinds of reduction operations are performed simultaneously and their outputs are combined as shown in Figure 13:

$$R_{a}^{k}=Concat\left\{Max\left\{X_{a}^{k}\right\},Sum\left\{X_{a}^{k}\right\},Avg\left\{X_{a}^{k}\right\}\right\}$$

where

$$X_{a}^{k}=\left\{T_{a,b}^{k}|b\in neighbor\left\{a\right\}\right\}$$

The reduced feature map is then combined with the input feature map of atom a (Figure 12) to produce the final output. This enables the model to obtain feature maps from different convolution levels, which are more straightforward and easier to optimize than only using the reduced feature map [19]:

$$A_{a}^{k+1}=Concat\left\{A_{a}^{k},R_{a}^{k}\right\}$$

In our experiments, the non-linearity f is the Leaky ReLU function with negative slope α=0.01:

$$f\left(x\right)=\{\begin{array}{c} x,\;if\;x>0\\ \;\alpha x,\;otherwise \end{array}$$

For each convolutional layer, a batch normalization operation [20] is applied on all atom embeddings of the entire batch to accelerate the training process.

### 4.3. Input Quantization

The initial input of the atom level features $A_{i}$ and pair level features $P_{ij}$ contains the entries listed in Table 3 and Table 4.

### 4.4. Multi-Task Learning

In ADME profiling in drug discovery, data sets of the same domain problem but different conditions, such as experimental settings, are usually found. For example, the aqueous equilibrium solubility of ligands in certain media (e.g., HCl) is correlated with those under different media (e.g., PBS), albeit they are not completely equivalent. A model targeting multiple related tasks will be much more powerful than independent models for each task.

**Figure 14:**
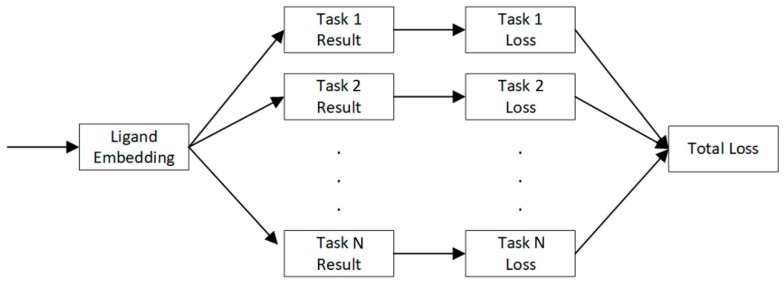
Joint training model for multi-task learning.

As shown in Figure 14 our MT-DNN model extends the single-task model in a joint learning setup. The embedding for each ligand is trained and then used to predict multiple-task scores simultaneously. When training, the loss functions of each task are summed to get the final loss function. Furthermore, the weight of individual tasks can be non-uniform. This is useful for scenarios which favor one task over other tasks.

### 4.5. Fine-Tuning

Due to the noisy nature of stochastic optimization algorithms, the validation and testing accuracy of neural network models varies greatly for each epoch. Hence, to obtain a stable model with consistent predictive power, some form of post-processing and model selection will be needed. In this paper, we provide a fine-tuning process, which combines model selection and ensemble to further improve the stability and performance of single-shot models.

**Figure 15:**
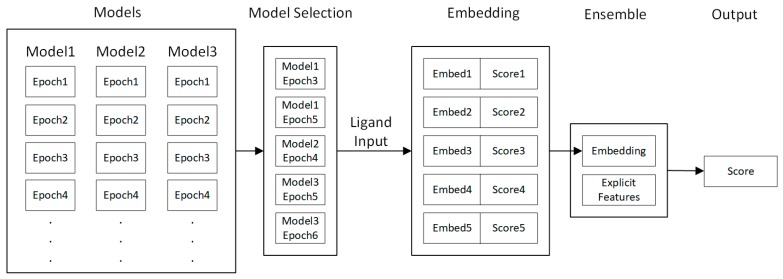
Fine-tuning algorithm.

The fine-tuning process works as shown in Figure 15. First, input data is trained by multiple network configurations consisting of different layer structures. Then, several of the best-performing models out of trained epochs of these models are selected based on their validation accuracy. Finally, the embeddings and prediction results of these models are used by the fine-tuning algorithm to train a fine-tuning model, which ensembles these embeddings and produces a model with improved accuracy.

The outputted embeddings and prediction scores for each selected model give the input of the fine-tuning model. The ensemble model consists of several multi-layer perceptrons. Order-independent reduction layers are also used to compress information from arbitrary number of models to a fixed size. After these embeddings and scores are transformed by the neural network, a final ligand embedding is produced. This embedding may be combined with an optional explicit feature vector to include any existing engineered ligand descriptors. The combined embedding is then transformed by a multi-layer perceptron to obtain the final predicted score.

### 4.6. Benchmark Method: Cubist

Cubist is a very useful tool in analyzing large and diverse set of data, especially data with non-linear structure-activity relationships (SARs) [21,22]. It is a statistical tool for generating rule-based predictive models and resembles a piecewise linear regression model [23], except that the rules can overlap. Cubist does this by building a model containing one or more rules, where each rule is a conjunction of conditions associated with a linear regression. The predictive accuracy of a rule-based model can be improved by combining it with an instance-based or nearest neighbor-based model. The latter predicts the target value of a new case by finding a predefined number of most similar cases in the training data and averaging their target values. Cubist then combines the rule-based prediction with instance-based prediction to give a final predicted value. Cubist release 2.04 was used in this study.

### 4.7. Benchmark Descriptors

Two-dimensional molecular descriptors were used for in silico ADME modeling. These include cLogP (BioByte Corp., Claremont, CA), Kier connectivity, shape, and E-state indices [24,25,26] of a subset of MOE descriptors [15], and a set of ADME keys that are structural features used for ADME modeling [27]. Some of the descriptors such as Kier shape indices contain implicit 3D information. Explicit 3D molecular descriptors were not routinely used in this study to avoid bias of the analysis due to predicted conformational effects and speed of calculation for fast prediction.

### 4.8. Data Sets

The test was performed on Amgen’s internal data sets using five ADME endpoints and a total of 13 data sets selected for building predictive model. The five selected ADME endpoints were human microsomal clearance (HLM), human CYP450 inhibition (CYP3A4), aqueous equilibrium solubility, pregnane X receptor (PXR) induction, and bioavailability. For the CYP3A4 assay, two subsets were studied, which differed slightly with conditions. For the aqueous equilibrium solubility assay, three subsets were studied: Hydrochloric acid (HCl), phosphate-buffered saline (PBS), and simulated intestinal fluid (SIF). For the PXR induction assay, six subsets were studied, which differed slightly with conditions. Across all ADME endpoints, the data sets used varied in quality and quantity. Generally speaking, PXR and bioavailability data sets were noisier than the data sets for the three other ADME endpoints.

The training set and test set were split in an approximate ratio of 80:20 (Table 5). To resemble real-time prediction situations, compounds were ranked with their registration data in chronological order. Newer compounds were selected in the test set.

### 4.9. Model Training and Test Procedure

The test set was used solely for testing purposes to avoid bias in the training procedure. The Caret package in R was used for the Cubist method. A 10-fold cross-validation was applied to tune parameters (committee member and number of nearest neighbors). A Caret-implemented grid search was then used to select the best parameter set to produce final models, using the lowest root mean-squared error (RMSE) for testing. For Chemi-Net, the input SMILES were first converted to 3D structures using an internal molecular conformation generator. The resultant molecular graphs were then used for training and testing. An MSE-based loss function was used for training the neural network. A standard neural network procedure using the Adam optimizer [28] was applied. Both the single-task and multi-task models were evaluated. The fine-tuning process was performed on all tests.

The Cubist benchmark calculation was performed in parallel on an internal CPU cluster. The Chemi-Net calculation was carried out on six Amazon Web Service (AWS) EC2 p2.8xlarge GPU instances.

## Figures and Tables

**Table 1:** Test set results.

Dataset	Subset	Train Size	Test Size	Cubist	Chemi-Net ST-DNN	Chemi-Net MT-DNN
HLM	1	69,176	17,294	0.39	0.445	
CYP450	1	3019	755	0.597	0.692	
	2	71,695	17,924	0.315	0.414	
Solubility	1 (HCl)	10,650	2659	0.493	0.548	0.585
	2 (PBS)	10,650	2664	0.393	0.471	0.498
	3 (SIF)	10,650	2645	0.445	0.552	0.562
PXR	1 @ 2 uM	19,902	4981	0.276	0.257	0.422
	2 @ 10 uM	17,414	4256	0.343	0.333	0.445
	3 @ 2 uM	8883	2223	0.094	0.11	0.199
	4 @ 10 uM	8205	2054	0.246	0.2	0.327
	5 @ 10 uM	10,047	2511	0.349	0.38	0.418
	6 @ 2 uM	10,047	2536	0.283	0.311	0.352
Bioavailability	1	183	46	0.115	0.123	

CYP450, cytochrome P450; DNN, deep neural network; HCl, hydrochloric acid HLM, human microsomal clearance; MT-DNN, multi-task deep neural network; PBS, phosphate-buffered saline; PXR, pregnane X receptor; SIF, simulated intestinal fluid; ST-DNN, single-task DNN.

**Table 2:** Performance of Chemi-net and Wenzel et al.’s method on publicly available data sets from the Wenzel study.

Task	Total Compounds	Training Set Size	Ext. Validation Set Size	Chemi-Net MT [R^2^]	Wenzel et al.’s MT [R^2^]
Human microsomal clearance	5348	4821	527	**0.620**	0.574
Rat microsomal clearance	2166	1967	199	**0.786**	0.783
Mouse microsomal clearance	790	734	56	0.325	**0.486**
Caco-2_Papp permeability	2582	2336	246	**0.560**	0.542

**Table 3:** Atom features.

Atom Feature	Description	Size
Atom type	One hot vector specifying the type of this atom	23
Radius	vdW radius and covalent radius of the atom	2
In rings	For each size of ring (3-8), the number of rings that include this atom	6
In aromatic ring	Whether this atom is part of an aromatic ring	1
Charge	Electrostatic charge of this atom	1

**Table 4:** Pair features.

Pair Feature	Description	Size
Bond type	One hot vector of {Single, Double, None}	3
Distance	Euclidean distance between this atom pair	1
Same ring	Whether the atoms are in the same ring	1

**Table 5:** Data set details.

Dataset	Subset	Train Size	Test Size
Human microsomal intrinsic clearance log_10_ rate) (µL/min/mg protein)	1	69,176	17,294
Human CYP450 inhibition log10(IC_50_ µM)	1	3019	755
	2	71,695	17,924
Solubility log10 (µM)	1 (HCl)	10,650	2659
	2 (PBS)	10,650	2664
	3 (SIF)	10,650	2645
PXR induction (POC)	1 @ 2 uM	19,902	4981
	2 @ 10 uM	17,414	4256
	3 @ 2 uM	8883	2223
	4 @ 10 uM	8205	2054
	5 @ 10 uM	10,047	2511
	6 @ 2 uM	10,047	2536
Bioavailability	1	183	46

CYP450, cytochrome P450; HCl, hydrochloric acid; IC_50_: half maximal inhibitory concentration; phosphate-buffered saline; pregnane X receptor; POC, percentage of control; SIF, simulated intestinal fluid.

## Supplementary Materials

Supplementary materials can be found at https://www.mdpi.com/1422-0067/20/14/3389/s1.
