# Supplementary material for: Chemi-Net: A Molecular Graph Convolutional Network for Accurate Drug Property Prediction
Source: Int J Mol Sci. 2019 Jul 10;20(14):3389. doi: 10.3390/ijms20143389 (PMC6678642; doi:10.3390/ijms20143389)
Supplement: Supplementary file 1 [file ijms-20-03389-s001.pdf]

Supplementary information

Dynamic batching algorithm

Most neural network models use stochastic gradient descent or one of its variants for optimization. Mini-batched inputs are generally favored instead of feeding individual instances one by one in order to reduce instance variance in stochastic optimization and computation overhead during training process. In most CNN models, the computation graph can be statically determined and is the same for all input data. In these cases, mini-batching can be easily implemented, as is done in most DNN frameworks. However, the structure of the graph defined by the CNN in this current study depends on the input molecules, thus it has highly dynamic and different computation graphs. Conventional neural network implementations fail to support batching on such a model because the computation graph is heterogeneous for each input. To overcome this issue with Chemi-Net, an efficient dynamic batching algorithm was designed. This algorithm analyzes the computation graph for each input batch dynamically and merges CNN operations without affecting the computation correctness. Additionally, unlike CNN implementations, which only supports batching at training instance level, our approach supports batching neural network operations in intra and inter instances. This is essential for a correct batch normalization implementation, and important for efficiently running heterogeneous convolution layers.

The detailed dynamic batching algorithm is shown in **Figure S1.** The algorithm can be divided into three steps: computation graph initialization, batching analysis, and gather/scatter operation generation. During initialization, the original un-batched computation graph for each input instance is generated. The computation graph is a directed acyclic graph (DAG). In the batching analysis step, the structure of the computation graph is examined so that compatible operations without data dependency are batched. In the gather/scatter operation generation step, gather and scatter operations are generated for the inputs and outputs of a batched operation. An optimization is also performed in this step so that a scatter-gather operation pair between two batched operations can be eliminated.

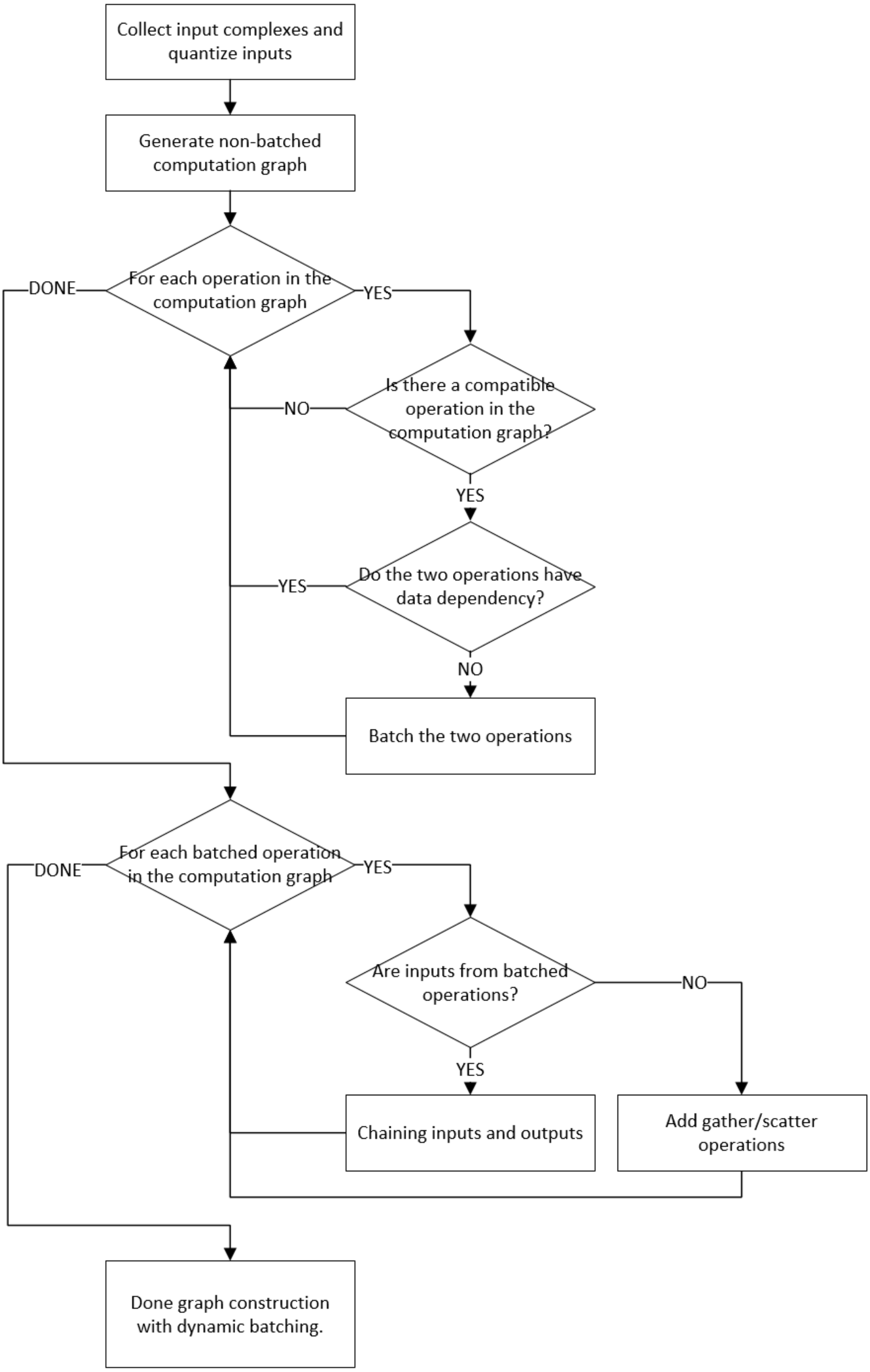

Figure S1. Dynamic batching algorithm.
